# The meaning of life according to patients with advanced lung cancer: a qualitative study

**DOI:** 10.1080/17482631.2022.2028348

**Published:** 2022-02-01

**Authors:** Jin Mei Zhang, Mei Rong Zhang, Chun Hong Yang, Yumei Li

**Affiliations:** a Department of Nursing, Shanghai Pulmonary Hospital, Tongji University, Shanghai, China; b Department of Respiratory Medicine, Hongkou Branch of Changhai Hospital, Naval Medical University, Shanghai, China; c Department of Nursing, Shanghai Yangpu District Mental Health Centre, Shanghai, China; d Department of Pediatrics, The First Affiliated Hospital of China Medical University, Liaoning, China

**Keywords:** Advanced lung cancer, meaning of life, qualitative research, terminal care

## Abstract

**Purpose:**

This qualitative study explores the meaning of life and end-of-life coping strategies among patients in China with advanced lung cancer.

**Methods:**

We conducted in-depth interviews with 21 hospitalized patients with advanced lung cancer and analysed the data using the 7-step Colaizzi method.

**Results:**

The analysis revealed themes in patients’ experiences and feelings about living with a terminal illness. These include: 1) The core of the meaning of life is “self-iteration,” which includes self-recognition and cherishing life; 2) The existence form of the meaning of life is “yu-wei,” including self-reliance and altruism; 3) The meaning of life is embodied in three levels: the past, present, and future. The past includes gratitude, guilt and remorse, and avoidance; the present includes using the support system, positive response, independence, and integrity; the future includes accompanying relatives, preparing for death, living a high quality of life, and worrying.

**Conclusion:**

Meaning of life is a multidimensional and diverse concept among patients with advanced lung cancer. Medical care providers and family members can provide targeted professional guidance and psychological support according to patients’ characteristics to help them discover their meaning of life, improve their quality of life, and achieve a positive end-of-life perspective.

## Background

According to global cancer statistics, in 2020 there were an estimated 19.3 million new cancer cases and almost 10 million cancer deaths worldwide (Sung et al., 2021). It is estimated that there were 4.5 million new cancer cases and more than 3 million deaths in China in 2020, accounting for approximately 23.8% and 30.2% of all cancer cases and deaths worldwide that year. In terms of incidence spectrum, lung cancer is the malignant tumour with the highest morbidity and mortality rate in China. In 2020, there were more than 810,000 new cases of lung cancer and about 710,000 deaths in China, accounting for 23.8% of all cancer deaths (He jie et al., 2021; Pan, 2021)

Lung cancer is one of the most life-threatening diseases worldwide. In the USA, among all cancer types, the five-year survival rate of lung cancer is 17.7%, while that of lung cancer patients in China is only 16.1% (De Guzman & Malik, 2019). Most patients with lung cancer are diagnosed at an advanced stage and usually receive complex treatment (Nasim et al., 2019), leading patients to experience more psychological symptoms, distress, fear of recurrence, and future uncertainty, which can seriously affect patients and their quality of life (Baudry et al., 2019; Chiou et al., 2016; Graves et al., 2007). The diagnosis and treatment of lung cancer can often disrupt many aspects of daily life, destroy self-cognition, attitude to life, personal assumptions, and value systems, and make the continuity and consistency of life lose meaning for patients. Furthermore, the side effects of the cancer treatment itself and its association with patients suffering from serious physical and psychological pain may cause feelings of helplessness, hopelessness, and a sense of meaninglessness.

Meaning of life (MoL) is crucial to achieving personal well-being in people with advanced disease (Mok et al., 2012). Finding the MoL is everyone’s mission, and it is a key factor in ensuring mental health and quality of life (Breitbart et al., 2010; Frankl, 1959 ; Henry et al., 2010; Mok et al., 2012). One’s understanding of individual survival and the idea that life varies from person to person changes over time, and may significantly change at specific times in one’s life; for example, with the diagnosis of a serious illness. Understanding MoL is a protective buffer for cancer patients and has been shown to relieve depression, reduce despair, and avoid accelerating death (Guerrero-Torrelles et al., 2017). It has been found that understanding one’s MoL is not only helpful in adjusting the life attitude and emotional response of patients after a cancer diagnosis, but also in coping with the disease more effectively (Van der Spek et al., 2014). For cancer survivors and patients approaching the end of life, happiness, tolerance of physical symptoms, depression, and despair can be improved by reconstructing the MoL, personal values, and how to live meaningfully and with dignity (Vehling et al., 2011).

The scales and questionnaires used in previous studies for MoL cannot fully capture the experience of MoL of patients with advanced cancer. In this study, qualitative interviews were used to understand more deeply, the MoL in patients with advanced lung cancer. Patients in the last stages of life, through positive psychological counselling, often develop a disease narrative, re-establish their priorities in life, find life’s purpose and hope, build a new self-awareness, and reformulate the meaning of their life (Zeng, 2019; Zhou et al., 2020).

## Methods

This qualitative study is part of a larger project to explore MoL, along with an intervention study of lung cancer patients.

### Design

This is an exploratory study that used a phenomenological-hermeneutic approach (Dreyer & Pedersen, 2009) to deeply understand the connotation of MoL in patients with advanced lung cancer, and to reveal the complexity of MoL and its impact on self. A phenomenological perspective attaches importance to rich contextualized descriptions based on experience, and helps the researcher to look at how the world is experienced by the participant by studying different aspects of consciousness and experience. This research dealt with experiences and meanings and intended “to capture as closely as possible the way in which the phenomenon is experienced within the context in which the experience takes place” (Hill et al., 2021, p. 466). Informants can illuminate the lived world, and our aim was to understand their psychological needs and provide guidance for professional intervention measures.

We followed the consolidated criteria for reporting qualitative studies (Tong et al., 2007).

### Participants

Advanced lung cancer patients were recruited from April 2018 to July 2018 using purposive sampling from a grade 3A hospital in Shanghai. We included patients who met the following eligibility criteria: (1) had advanced (stage 3 or 4) lung cancer diagnosed by pathological examination; (2) was aged ≥18; (3) was able to provide written informed consent and able to understand questions and express themselves clearly; (4) was willing to participate in the study; and (5) had a Karnofsky score ≥ 60 (i.e., most activities of daily life could be done independently, with an occasional need for help). The exclusion criteria were as follows: (1) psychiatric disease (or history of psychiatric disease); and (2) inability to complete the questionnaire because of severe psychological distress, cognitive impairment, or visual disability.

A total of 21 participants, including 5 women and 16 men, between 32 and 73 years old (M = 58.51; SD = ±8.73), were included in the study. The key characteristics of the participants are detailed in Table I.

**Table I.** Participant characteristics (*N* = 21)

Characteristics		*n* or (M ± SD; range)
Age (years)		58.51 ± 8.73; 32–73
Gender		
	Male	16
	Female	5
Marital status		
	Single	2
	Married	16
	Divorced	2
	Widowed	1
Religion		
	Christian Church	3
	Taoism	1
	Buddhism	2
	None	15
Educational level		
	Primary	1
	Junior high	8
	Senior high	4
	University	3
	Other	5
Duration of disease		
	≤6 Months	6
	6–12 Months	5
	≥12 Months	10

M = Mean; SD = Standard deviation.

### Data collection

The first author conducted all face-to-face interviews (Table II) from April 2018 to July 2018. We conducted individual semi-structured interviews with open-ended questions to capture MoL in patients with advanced cancer after diagnosis. A preliminary semi-structured interview outline was developed based on a literature review and a group discussion with lung cancer specialists. Three patients were interviewed prior to the main study to determine the efficacy of the interview outline (these interviews were not included in the analysis). The interview guide was modified iteratively, as the interviews and concurrent data analysis proceeded, to incorporate new information and to focus progressively on emerging themes.

**Table II.** Interview guide

Number	Interview guide	Interview time
1	How has your attitude towards life changed since your diagnosis?	30–40 min/time
2	What impact have past suffering and setbacks had on you?	
3	What do you think is most important to you or your top priority?	
4	If you could have your life over again, how would you live it?	
5	How do you understand MoL?	
6	In retrospect, if there was one thing that could do to improve the meaning of your life, what would it be?	
7	What do you want to do about the future? What are your goals?	
8	What do you think of death?	

A total of 50 interviews were conducted, with an average of 2.5 interview sessions per patient to clarify the patient’s thoughts. The interviews were recorded using a recording pen, digitally stored, and then transcribed by the interviewers. Interviews ranged in duration between 30 and 40 minutes, during which the interviewer kept field notes that were referred to during the analysis. If the patients felt tired, the interview was stopped and either another appointment was made to continue the interview or the patient took a break to rest.

### Data analysis

Within 24 hours after the end of the interview, the recorded data were converted into text and imported into QSR Nvivo 8.0 software for sorting and analysis. To analyse the data, we used Colaizzi’s 7-step analysis (Zheng & Yu, 2018). Initially, all the authors read the transcribed material, searching for meaning and patterns. To ensure the authenticity, accuracy, and credibility of phenomenological research, researchers should analyse the data they collect in 7-steps (Colaizzi, 1978). In recent years, more qualitative researchers—especially those in the nursing field—have adopted the “Seven steps of Colaizzi Phenomenological Research Data Analysis” in data analysis methods (Liu, 2019).

The method reports patterns within qualitative data using 7 steps: familiarization with the data, identifying significant statements, generating initial codes, clustering themes, developing an exhaustive description, defining and naming themes, and seeking verification of the fundamental structure. If there is a deviation, the researcher must start the analysis step-by-step from the first step. For a detailed description, see Table III.

**Table III.** Colaizzi phenomenological analysis of seven steps and description

Steps	Description
1. Familiarization	Through repeated and careful reading of the collected data, the researcher became fully familiar with and understood all the information provided by the participants.
2. Identifying significant statements	We extracted meaningful statements that are consistent with the phenomena studied;
3. Formulating meanings	Researchers construct/encode the meanings of recurring ideas, bracket our own existing phenomenon-related presumptions as possible.
4. Clustering themes	Searching for common concepts to form themes, subject groups, and categories. At this point, you still need to “bracket” our existing ideas or experience, especially theoretical knowledge from the literature.
5. Developing an exhaustive description	The researcher should provide a detailed description of each topic generated in step 4 and may extract and add references original statement from the participants.
6. Producing the fundamental structure	Repeat comparisons of similar themes and their descriptions to identify and extract similar perspectives; Then construct a short and intensive phrase, the subject.
7. Seeking verification of the fundamental structure	The resulting topic structure is returned to the study participants for verification, asking whether their real experience has been captured to ensure the accuracy of the results. If there is a deviation, the researcher must start the analysis step by step from the first step.

We met the criterion of credibility through open-ended questioning and by providing a detailed description of the method that was followed. Each transcription was independently read, checked, and coded by two of the authors. We reached final interpretations through an agreement among all four authors. To meet the criterion of confirmability, we presented rich quotes from the participants that depicted each theme (Hill et al., 2021). All the authors who performed the analysis were engaged in clinical tumour nursing, particularly lung cancer care, and psychological nursing. One of the researchers has psychological consulting qualifications.

### Ethical considerations

This study’s procedures adhered to the regulations stipulated in the Helsinki Declaration (World Medical Association [WMA], 1964/2013) and was conducted in accordance with the Ethical Standards and Procedures for Research that was approved by the Ethics Committee of the Shanghai Pulmonary Hospital, Affiliated to Tongji University, project number: K20-252.

All patients were interviewed by the first author, who was familiar with the disease, but not with the participants. The interview was conducted in a meeting room of the ward. The environment was quiet and undisturbed. At the outset of each round of interviews, the researcher reminded participants of the associated nature and procedures of the research study, allowing participant’s time to discuss with others and consider options. During the interview, the patient’s right to privacy and confidentiality was respected, and all information obtained from the study subjects was strictly confidential. In all transcriptions, letters and numbers were used in place of real names.

## Results

**Figure 1.**
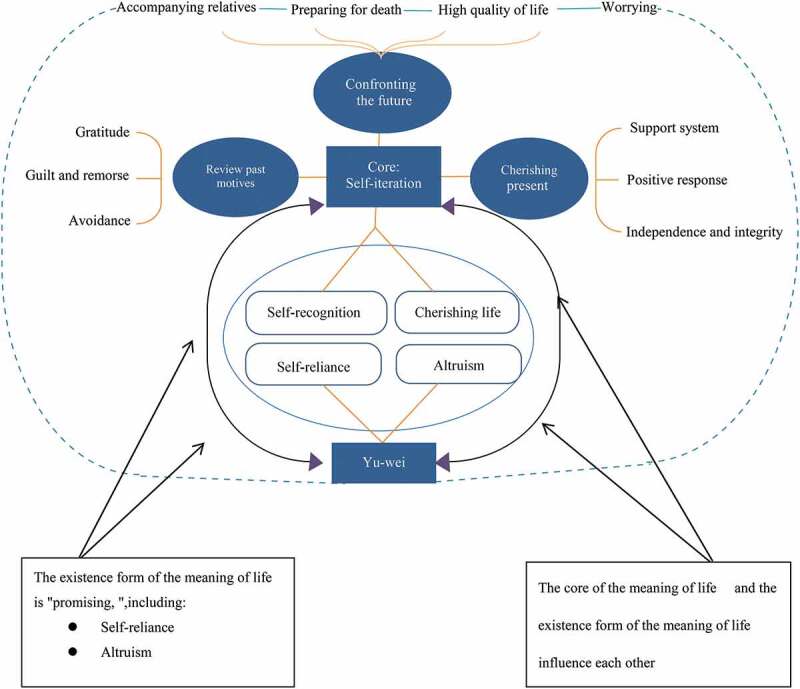
Connotation composition of meaning of life in patients with advanced lung cancer.

After analysing the interview results, we extracted one core aspect of MoL, two existence forms of MoL, three levels, seven themes, and ten sub-themes. The composition of MoL for patients with advanced lung cancer is shown in Figure 1.

### The core of MoL is self-iteration

#### Self-recognition

When most patients are told that they are diagnosed with advanced lung cancer, the first reaction is “impossible” or “wrong.” However, they then slowly accept the reality and follow the doctor’s treatment. Patients repeatedly think: “Why me?” “How long can I live?” “How do I live longer?” and so on. After a period of psychological struggle following the diagnosis, some patients start the process of self-recognition, also described by Stanze et al. (2019). The analysis shows that the experience of lung cancer is accompanied by self-recognition. With self-reflection, patients searched for things they had done to cause their illness, and were extremely determined to change bad habits and think deeply, to make themselves better and face the future calmly. Therefore, patients develop (action) strategies to cope with disease-related challenges.

After careful reflection, it must be because my previous lifestyle was not good. I often stayed up late, smoked, drank alcohol, and rarely exercised. After illness, I began to change my lifestyle by quitting smoking and drinking, running, and exercising every day instead of staying up late. (P1, male, 58)

It seems that I did not know myself before. With the continuous chemotherapy and radiotherapy after my illness, I began to think about why I was sick and how I should live in the future. Now I have a goal in life; I want to be a good husband, son, and father, which I did not do well before; I should plan my future life well. (P13, male, 54)

#### Cherish life

Under the threat of illness, most patients begin to think about life, the meaning of their life, and what to do in the face of illness in new ways. Most patients choose to deal with life events with a positive attitude, cherish life more, use the limited time with their families, do some meaningful things, and pay more attention to their health.

Life is limited, we must live well in the days to come, cherish the rest of time, cherish life, and enjoy family. I used to go on business trips and spend little time with my family. I feel regretful when thinking about the present situation. (P19, male, 59)

I think life is precious but cannot be forced. Sometimes we should cherish it, and when it is no more, let it go. Illness is inevitable in life. (P10, male, 67)

### The existence form of MoL is “yu-wei”

The existence form of MoL is “yu-wei,” which means “to do something or treat something,” and contains two themes: “self-reliance” and “altruism.” The participants in this study could take care of themselves and fulfill their daily life roles independently, but doing things for others makes one feel worthy of existence. The value of life needs to be measured in depth, rather than in time. The value of a person to society, first, depends on his feelings, thoughts, and actions that play a role in promoting the interests of human society.

#### Self-reliance

The self-reliance expressed by patients mostly referred to the ability to take care of themselves in daily life and do what they please.

It is difficult for a person who has never been recognized to develop enthusiasm for life. That is why we work hard, not only to strive for a better life, but also to become a recognized member of the group and realize ourselves. After my diagnosis, my family took good care of me, which seemed to make me lose the ability to take care of myself. I don’t think so. What I can do must be done by myself, and after my discharge I will return to work. (P5, male, 32)

#### Altruistic

Patients expressed the importance of altruistic behaviour, which referred to doing things for others and society.

After illness, I found that I like my job so much and I can do something for society by teaching. That is the meaning of my life (smile). (P5, male, 32)

Life should be like a candle, burning from the top to the bottom, always bright, only dedicated to society; with serious and hard work, can we find the meaning of life. My greatest happiness is seeing my patients recover, and I think that’s what I exist for. (P20, female, 56)

### Cherish the present life

Most of the patients expressed positive feelings towards the present. Many factors contributed to their positive attitude and motivation, such as using support systems and actively coping with the disease and difficulties in life. They also expressed the need for independence and integrity, stating that maintaining independence and integrity is closely related to the quality of life. Patients proposed maintaining their role in society and status to better understand MoL.

I never thought about the meaning of life before. Since life is limited, in the few days I have left I must live well, cherish the rest of my time, and enjoy life with the family. I used to go on business trips and spend more time away from home. I feel regretful when I think of the situation now. (P3, male, 66)

#### Support systems

##### Family support

All support is valuable, and the support of relatives is vital to patients. Family support involves elements such as parents’ encouragement and sponsorship, close relationships, harmony within the family, non-rebuking attitudes, and material support. When receiving a cancer diagnosis, the patient perceives the support from the family, who talks about the situation and shares feelings. Almost all the respondents experienced emotional support and practical support at home and in other activities.

Since my illness, the focus of the whole family has shifted to my treatment. It is the support of my family that encourages me to fight against the disease. They help me buy medicine and find doctors. Really, if it were not for them, I would not have recovered so quickly and well. (P3, male, 58)

All the family are very concerned about my illness; the children help me to go around for expert consultations and get active genetic testing. Actually, I love to be sick. When I was hospitalized, my wife always accompanied me and took care of me. I feel very happy. (P8, male, 63)

##### Medical support

Healthcare professionals, especially nurses, play an important supporting role during illness and treatment. All the patients interviewed mentioned that they thought the healthcare professionals were very skilled and experienced in the treatment of advanced lung cancer, which made them feel safe and confident in the treatment.

The results of my regular review after discharge, the occurrence of drug reactions and colds, etc., can all be obtained through the outpatient service in a timely manner. Sometimes call doctors and nurses can also reply in a timely fashion, and I often receive surveys from the hospital to get my feedback about the hospital. All these make me feel that medical treatment is at hand and I should choose active treatment to face the disease. (P15, female, 58)

##### Organizational support

Organizational support in our study refers to support from organizations or governments, including material assistance and direct services. The impact of organizational support on physical health is significant for individuals experiencing high levels of stress or stressful events.

The employee welfare system of our unit is very good. Some examination and treatment fees can be reimbursed for me, which lightens my financial burden. I want to thank the unit for their support. (P6, male, 58)

I joined the Cancer Society’s national meeting, which is a patient-to-patient meeting. They are friendly and often give me support, and sometimes they talk about the latest treatment progress and drug research together. Through contact for half a year, I solved a lot of psychological confusion. I really appreciate the support of my companions, which is very helpful to me. (P4, male, 61)

##### Positive response

Patients’ positive response refers to them cherishing life. For some patients, it means actively seeking treatment and participating in various anti-cancer organizations. Others actively participate in clinical research on new drug trials, hoping to create an opportunity to extend their lives.

I bought a lot of books on lung cancer treatment, and I read almost all of them, gained a lot; all kinds of treatment have complications you need to pay more attention to, for example, it is very important to pay attention to the prevention of colds after radiotherapy. (P18, male, 59)

#### Independence and integrity

Patients with advanced lung cancer reported experiencing the freedom to pursue inner spiritual integrity and an independent self.

Disease is disease: I am just in a special stage of my life. Every time I take a week off from chemotherapy, I drive to the suburbs hoping to find comfort in my return. (P13, male, 54)

It is difficult for a person who is never recognized to be enthusiastic about life, so we work hard, not only to strive for a better life, but also to be a recognized member of the group and to realize ourselves. (P3, male, 66)

### Confronting the future

All the patients expressed that they were looking forward to the future and hoping to have time to enjoy life. Although life was limited, there was still a lot to do, and they hoped to make a good plan and reasonable arrangements to do what they could. Some patients wanted to participate in family development and spend their limited time with their families, some patients said they are not afraid of death, or have never thought of it, some patients had quietly made their own arrangements for death, some patients worried that their death would bring sadness to their loved ones, and some patients avoided talking about death entirely.

### Time with loved ones

All participants wanted to be with their loved ones in the later stages of life. They believed that the meaning of their lives is to accompany their families and bring them happiness. When the patients knew that they were getting closer to death, most were filled with suffering, loneliness, and fear. At this time, only the company of family could alleviate this fear and psychological pain.

I am very satisfied with watching TV and chatting with my family in the limited time. I don’t want to go to the hospital anymore. (P15, male, 70)

I didn’t delay work after I was ill, accompany my wife and daughter after work, my family supported me very much, and being with them was the happiest time for me. (P11, female, 51)

#### Preparation for death

The fear of death is a fundamental source of anxiety. Most of the interviewees said that when they were told of their diagnosis, the first thought was that they had received a prison sentence and were close to death. There is a fear of death, depression, worry for the family, hope to prolong life through active treatment, and resistance to talking about death. Many participants slowly adapted to the diagnosis and shorter life expectancy. There were also patients who were ready to die. The attitudes of most of the older patients were calmer, not afraid or worried.

Living to be 100 or 50 is the same thing. It is like riding a train. Some get off at the terminal and some get off halfway. I was not afraid I was going to die when I got sick, there was so much to do (touch the head). (P4, male, 61)

#### High quality of life

Good patient compliance and aggressive treatment are aimed at controlling disease progression and improving quality of life. All interviewees expressed their wishes for a high quality of life, and hoped to make full use of their limited time to live a better life. All the patients said that they hoped to go out for a walk and experience nature after their condition stabilized. Some patients had carefully planned different activities.

I am going to retire soon. I planned my life after retirement. If my condition is stable, my husband and I will travel to enjoy the beautiful scenery of our great motherland. (P19, male, 59)

#### Worrying

Almost all interviewees experienced uncertainty and anxiety during treatment, and most patients expressed worry about not being able to predict disease progression, thought that their death would bring emotional pain to their families, and a few patients expressed that they worried about their families’ later lives, due to costs related to medical treatment and the loss of the family’s main income. The support of relatives is very important, but at the same time, the patients were very sensitive to the response of their relatives. They worried about how their children or elderly parents would react when they found out about their illness, which caused them a lot of emotional distress and psychological pain.

I have been adhering to the treatment until now, but I cannot see any effect. There is bone metastasis and pain again, which makes me worry. One day I will leave suddenly, and my loved ones will be very sad. (P1, male, 58)

My parents are old, and their financial resources are limited. The kids need tuition and home loans; my family’s financial situation will be a big problem in the future. (P11, female, 51)

### Review of the past

#### Gratitude

Gratitude can help improve the sense of life, and in reviewing their lives, the interviewees expressed their gratitude for the help they had received, particularly due to national policies regarding medical insurance.

After receiving medical insurance for serious illnesses, many expenses can be reimbursed. People benefit from the national policy. Now, the cost of some targeted drugs can also be included in medical insurance, and ordinary people can save a lot of money every month, thanks to the Party and the state. (P14, male, 60)

#### Guilt and remorse

In the face of complicated negative emotions such as anxiety, depression, helplessness, and fear, the discussion of reviewing the past mainly focused on guilt and remorse; a diagnosis of lung cancer often has a strong relationship with a sense of shame (Margetic et al., 2020). Half of the interviewees had a history of smoking, felt guilty about their unhealthy lifestyle, and often blamed themselves. They feared that if people around them knew they have lung cancer, their relatives would be discriminated against; such fear of stigmatization is higher in the Chinese culture than in Western cultures. Some of the interviewees said they felt guilty about being taken care of by their families.

I have been smoking for many years, but I didn’t know it was not good until I fell ill with lung disease. I stopped going to parties with friends to avoid being judged by others. Now I have given up smoking. (P15, male, 57)

I stopped hanging out with friends for fear that they would ask about the illness, I would be looked down upon or suspected of being contagious. When I went to a family reunion during the Spring Festival, I bought my own tableware so as not to cause trouble. (P16, male, 60)

#### Avoidance

The interviewees refused to talk about certain topics. Because of illness, they often avoid social contact and were unwilling to meet and talk with acquaintances. As lung cancer is often perceived as being a result of smoking or unhealthy lifestyle (Yang et al., 2021), some respondents chose to avoid telling their friends and others about their illness. They tried to cover up their symptoms and act as usual. Nababan et al. (2020) point out that lung cancer patients avoid talking about their situation because they think it would be a burden for their family and friends.

I have been visiting my parents regularly, but I don’t talk about my illness with my friends and other relatives. (P13, male, 54)

## Discussion

This study posits that lung cancer can be perceived as a highly threatening disease that changes one’s self-recognition and starts patients thinking about the meaning of life. Patients experience a sense of uncertainty about the future after diagnosis (Dönmez & Johnston, 2020). They fear death, and therefore avoid discussing the disease. They also feel shame and guilt due to their previous unhealthy lifestyles and the burden that their illness has brought to their families. After a period of reflection and adaptation, however, they gradually experience an awareness and acceptance of dying when nearing the end of life (Arantzamendi et al., 2020; O’Gara et al., 2018) and take various measures to cope with the challenge of the disease. Regarding the patients’ needs, greater emphasis must be placed on psychosocial care as part of the biopsychosocial model to adequately address the patients’ concerns.

### Helping the patient to adapt to the new self through reflection and change to realize the unity of the physical, mental, and spiritual health

The MiL is difficult to understand or describe. This research used the method of interpretive phenomenology while interacting with patients to help patients analyse the longitudinal life axis, encourage them to share experiences, and emphasize understanding, using their own words to express their thoughts about the world and their emotions (Costas-Muñiz et al., 2019; Tsai et al., 2020). We attempted to help patients realize their MiL, engage in self-recognition, constantly improve themselves in terms of behavioural change and positive adjustment of mood for spiritual satisfaction, and self-iterate through meaningful labour and creativity, which enhances self-worth, thereby enhancing the sense of MiL. The exploration of MoL cannot be completed independently; it requires the help of a support system and the guidance of professionals to help individuals realize the unity of physical, mental, and spiritual health from a broad spiritual perspective (Edwards et al., 2010).

### Establishing a support system to increase patients’ sense of understanding, management, and control of the disease

A study by Maliski et al. (2003) show that the family is an important source of emotional support and assistance. Patients want to maintain their independent and complete family roles by living as they did before their illness. Consistent with the results of Nieto-Guerrero Gómez et al. (2020), participants in the current study wanted to maintain a normal life and not depend on their families. Patients who experienced positive social support have been shown to be more likely to deal with disease-related stress and distress, have reduced anxiety and depression, adapt more effectively, and live longer (Golden et al., 2020). The respondents in this study expressed the importance of participating in social activities and maintaining their role in society. They had plans for their futures, regardless of whether they were short or long. Increasing hope has been shown to help patients lead a normal life (Maliski et al., 2003), experience a meaningful life, and carry out creative work (Guan et al., 2021).

In this study, interviewees talked about the network of their support staff, but also stated that they had encountered difficulties in seeking help and relying on others. They wanted to protect and reduce the burden for their relatives and friends. Similar results were reported by Tavares et al. (2020).

### Early psychological intervention to reduce negative emotions and establish a positive attitude towards life

The treatment of advanced lung cancer is a long-term and continuous stressor, and various complications cause great physical injury and psychological impact on patients. Most patients with advanced lung cancer feel negative emotions (e.g., anxious, depressed, afraid, hopeless, etc.), have difficulty adjusting, and feel uncertain about the effect of treatment. Individualized psychological intervention is necessary to help patients maintain hope, which is an important factor in managing one’s life. Medical resources are an important source of hope (Luoma & Hakamies-Blomqvist, 2004). Support and encouragement from healthcare providers can increase hope, reduce uncertainty about the illness, improve patients’ ability to cope, and improve their quality of life (Guerrero-Torrelles et al., 2017). Living in the moment is considered an effective coping strategy for reducing worries in people approaching the end of life (Dönmez & Johnston, 2020). Nursing through having conversations with patients about their hobbies or daily lives, talking with them about issues that are non-illness related, encouraging them to partake in various activities and discussing with these patients the most important things in their lives, helps them to find meaning in life and to live in the moment .

### Education on death to reduce fear of death

The traditional Chinese view of death is to enjoy life and hate death, and there is a prevalent negative attitude of denying, covering up, and not accepting death. In contrast, Western culture has a more rational and open-minded understanding of death, and can face death directly (Bai & Yin, 2014). Although it has been reported that the application of systematic death education in palliative care can reduce anxiety and depression and play a positive role in improving patients’ quality of life, death education in China is still in its infancy (Hangting et al., 2020; Temel et al., 2017). In this study, most patients felt a sense of crisis or fear related to death, brought about by their diagnosis; they simultaneously cherished their lives, actively took part in their cancer treatment, and hoped to extend their lives. However, the end of life cannot be separated from death. When talking about death, interviewees said they were not prepared to talk to their relatives about it, for fear that they would not accept the situation. Medical care providers can help patients understand their disease and guide them to conduct regular self-assessments of their physical abilities to understand their own conditions and reduce unnecessary worries. At the same time, these professionals with psychological training should use their skills to carry out death education regularly to discuss the value and manifestation of life, namely “life” and “death,” from the perspective of the MoL. With the aid of religious beliefs and social cultural forms, patients can increase their awareness and acceptance of dying. Research shows that people with active religious beliefs cope better with death and the pain of death (Mohammadzadeh & Najafi, 2020). In February 2019, the National Health Commission issued a notice of the General Office of the National Health Commission on the implementation of the pilot work of “Internet + Nursing Service” (Yang & Meng, 2019). It is necessary to focus on providing health education, hospice care, and other nursing services for end-stage patients, thus providing an opportunity for online death education for communities and families.

### Strengths and limitations

To ensure full reporting on all relevant matters and to enhance the trustworthiness and transparency of the results, we strictly followed the guidelines for qualitative research (Tong et al., 2007). We also meticulously described the step-by-step analysis and quotes from the participants to enhance trustworthiness and transparency.

Although there have been many quantitative studies conducted on MiL in patients with lung cancer (Zeng, 2019; Zhou et al., 2020), hardly any qualitatively empirical studies exist on the experiences and MoL related to advanced lung cancer at the end of life from the patients’ perspective. This is the first Chinese study focusing on this patient group; therefore, it makes a unique contribution to the literature.

A limitation of this study is that patients were recruited from only one hospital in Shanghai. China. However, the hospital admitted lung cancer patients from all over the country. A second limitation is that this study included 21 patients in total. The sample was nevertheless sufficient to reach data saturation, meaning that the findings should be generalizable. In addition, only 5 women, compared to 16 men participated; this is because male lung cancer patients account for a higher proportion in the population. It is uncertain whether gender will affect the results of the study, and further research on this aspect is required in the future.

## Conclusion

This study focused on patients’ self-reported understanding and feelings regarding MoL. It explored the life attitudes, self-worth, and coping strategies of patients with advanced lung cancer in the face of a life-threatening illness, revealing the multidimensionality of MoL and its impact on self from multiple dimensions. The findings of this study can guide nurses and other healthcare professionals to strengthen the psychological and social assessment of patients in clinical nursing and understand their psychological state and needs. This includes psychological intervention and counselling, a family-centred nursing mode, and multidisciplinary collaborative nursing from the body-mind-spirit and other multidimensional areas to meet the needs of patients’ treatment and rehabilitation. This could help patients to understand the meaning of existence and past setbacks, and help nurses to apply dedication and action to develop strategies for improving patient-centred care in China and to enhance the sense of MoL.

## Data Availability

All data, models, and code generated or used during the study appear in the submitted article.

## Geolocation information

Shanghai Pulmonary Hospital, Tongji University, No. 507 Zheng Min Road, Shanghai, China.
